# Household Hints and Recipes

**Published:** 1877-10

**Authors:** 


### Household Hints & Recipes.

To RESTORE GILT.—Ammonia and water will often restore French gilt, if not too much worn off.

To REMOVE PRINTING INK FROM ANY ARTICLE.—Printing ink can be removed from any article by the use of ether, turpentine, or pure benzine.

OFFENSIVE SMELL IN THE FEET.—Bathe them in a weak solution of permanganate of potassa; one scruple of the salt to eight ounces of water.

To KEEP MEAT FRESH.—Suspend the meat in a close vessel, on the bottom of which some strong acetic acid has been poured. In this way the meat may be kept fresh for a considerable time.

CURE FOR SOFT CORNS.—Dip a piece of linen rag in turpentine and wrap round the toe on which the corn is situated, night and morning. The relief will be almost immediate, and in a few days the corn will disappear.

FRENCH BLACKING. — Twenty ounces ivory black, sixteen ounces New Orleans molasses, five ounces linseed oil, one ounce sulphuric acid, one-quarter ounce indigo, one-half ounce mucilage; mix the black and molasses well, so as to get out all the lumps; next add the oil; then by degrees the acid, and as much water as may be required to form a paste.—*Druggists' Circular.*

Tasteful ornaments may be made of natural leaves and sprays artificially frosted. This is done by means of powdered glass, which can easily be obtained by pounding some bits of glass with a heavy hammer, care being taken to protect the eyes against flying splinters. Dip the objects in thin gum water and shake the powdered glass over them, when handsome bouquets can be arranged.—*Druggists' Circular.*

To REMOVE TIN FROM TINNED COPPER.—For various reasons, it is occasionally expedient to remove the tin from tinned copper vessels or utensils. Professor Boettger recommends for the purpose a solution of sesquicloride of iron. The vessel to be cleaned is filled with it or immersed in it, and in a few minutes, according to the thickness of the tin, it will be entirely removed, and it is only necessary to polish the copper with sand slightly moistened with very dilute hydrochloric acid.

To CLEAN STRAW MATTING.—Wash it with weak salt and water, and dry it well, or boil a small bag of bran in 2 gallons of water, and wash the matting with the water, drying it well.

To RELIEVE HARD CORNS.—Bind them up at night with arnica, to relieve the pain. During the day, occasionally moisten the stocking over the corn with arnica, if the shoe is not large enough to allow the corn being bound up with a piece of linen rag.

To REMOVE STAINS FROM WHITE MARBLE.—Take 1 oxgall, 1 wine glass soap lees, ½ wine glassful turpentine; mix and make into a paste with pipe clay. Put the paste on the stain and let it remain for several days. If the stain is not fully removed a second application will generally prove sufficient.

To FIX INK.—A simple method of rendering paper so retentive of ink that the latter cannot be removed without leaving plain marks has been lately adopted in France for checks. The main feature of the method is the passing the paper through a very weak solution of tannic acid in distilled water.

CARBON BISULPHIDE LAMP.—Eighteen or nineteen years ago, writes Joseph Coupland, in the *Pharm. Journal*, in investigating the effect of burning spirit solutions of several essential oils in the ordinary perfume lamp, with spongy platinum ball, I extended my experiment to solutions of chloroform, iodoform, bromoform and bisulphide of carbon, with results which encouraged the expectation of a practical application of the method for disinfection. From a commercial point of view the cost and limited use of so elegant and convenient a method induced the making of more experiments, and resulted in realizing the burning of solutions of chloroform, iodoform and sulphide of carbon in the ordinary methylated spirit of commerce in an ordinary spirit lamp, for which I afterwards substituted a lamp made especially for me by the York Glass company, entirely of glass and glass wick-holder with cup.

I have employed this mode of generating disinfecting gases on my own premises, and also for disinfecting rooms where contagious diseases had occurred, and can recommend what is comparatively a cheap plan, as well as easy of application.

The suggestion of Mr. Groves is a very good one, as insuring safety from breakage, and I have no doubt that a special lamp would find a ready sale, if produced at a moderate cost.

TO CLEAN CANE-BOTTOM CHAIRS.—Turn up the chair bottom, and with hot water and a sponge wash the cane-work well, so that it may become completely soaked. Should it be very dirty you must add soap. Let it dry in the open air if possible, or in a place where there is a thorough draught, and it will become as tight and firm as when new, provided it has not been broken.

---

TO CURE HAMS.—Cover the bottom of the cask with coarse salt, lay on the hams with the skin side down, sprinkle over fine salt, then another layer of hams, and so on until the cask is full. The cask should hold from 64 to 120 gallons. Make a brine as follows: 6 gallons water, 9 pounds salt, 4 pounds brown sugar, 3 ounces saltpetre, 1 ounce saleratus. Scald and skim, and when cold pour the brine into the cask until the hams are completely covered. Let the hams remain in this pickle for three months, a little longer would do no harm. A handful each of mace and cloves scattered in the brine will improve the flavor of the meat.

---

TO CURE BEEF AND PORK.—To each gallon of water add 1½ pounds salt, ½ pound sugar, ½ ounce saltpetre, and ½ ounce potash. Boil these together until the dirt from the sugar rises to the top and is skimmed off. Then put it into a tub to cool, and when cold, pour it over the beef or pork, to remain four or five weeks. The meat must be well covered with pickle, and should not be put down for two days after killing, during which time it should be slightly sprinkled with powdered saltpetre, which removes all the surface blood, &c., leaving the meat fresh and clean. Ham cured in this way may be smoked as usual, and will be found excellent.

---

CONSERVATORY ON THE HOUSE-TOP.—That excellent plan which we have so often advocated, of turning the tops of the houses in cities into gardens, has been carried ont by the Palmer House in Chicago, and a portion of the roof of that hotel is now covered with a magnificent conservatory. The structure is entirely of glass and iron, and as it is built on an extension, its location is such that it opens directly out of the fifth floor corridor of the main edifice, which rises some two stories above. A fine collection of tropical and rare plants has been provided, and the regular heating apparatus of the house supplies ample warmth. The conservatory is open to guests of the hotel, and furnishes a delightful resort.—*Scientific American.*

A CURE FOR SMALL POX.—"I am willing to risk my reputation as a public man," wrote Edward Hine to the Liverpool *Mercury*, "if the worst case of small pox cannot be cured in three days, simply by the use of cream of tartar. One ounce of cream of tartar dissolved in a pint of water, drank at intervals when cold, is a never failing remedy. It has cured thousands, never leaves a mark, never causes blindness, and avoids tedious lingering."

---

TO CLEAN OIL PAINTINGS.—Put into 2 quarts of strong lye, ½ pound of Genoa soap, rasped very fine, with 1 pint spirits of wine; let them simmer on the fire for half an hour, then strain them through a cloth. Apply the preparation with a brush to the picture, wipe it off with a sponge, and apply the second time, which will remove all dirt. Then with a little nut-oil warmed, rub the picture and let it dry. If the canvas is injured by damp, mildew or foul air, the first thing to be done is to stretch and line it with new canvas.

---

TO CLEAN PICTURES.—Having taken the picture out of the frame, take a clean towel, and, making it quite wet, lay it on the face of the picture, sprinkling it from time to time with clean soft water; let it remain wet for two or three days; take the towel off and renew it with a fresh one. After wiping the picture with a clean wet sponge; repeat the process till you find all the dirt is soaked out of it; then wash with a soft sponge, and let it get quite dry; rub it with some clear nut or linseed oil, and it will look as well as when freshly done.

---

TO CLEAN PAPER HANGING.—Cut into 8 portions a loaf of bread 2 days old; it must neither be newer nor staler. With one of these pieces, after having blown off all the dust from the paper to be cleaned, by means of a good pair of bellows, begin at the top of the room, holding the crust in the hand, and wiping lightly downward with the crumb, about half a yard at each stroke, till the upper part of the paper is completely cleaned all round. Then go round again, with the like sweeping stroke downwards, always commencing each successive course a little higher than the upper stroke had extended, till the bottom be finished. This operation, if carefully performed, will frequently make very old paper look almost equal to new. Great caution must be used not to rub the paper hard, nor to attempt cleaning it the cross or horizontal way. The dirty part of the bread, too, must be continually cut away, and the pieces renewed as soon as may become necessary.

DESTRUCTION OF VEGETABLE GROWTH, MOSSES, ETC., ON WALLS.—If you wish to clean a wall of mosses, lichens, fungi, etc., says a scientific contemporary, mix a solution of one per cent. carbolic acid, and the remainder clean water, and give the wall a coat (if it may so be called) with a brush. An hour or two afterwards the dead vegetation may be washed off with clean water.—*Druggists' Circular.*

---

CHLORIDE OF LIME AS A DISINFECTANT.—One pound requires three gallons of water; use the clear solution. To purify rooms, sprinkle on the floor, and, if needful, on the bed-linen. Infected clothes should be dipped in it, and wrung out, just before they are washed. It purifies night commodes, water closets, &c. It may also be used in its pure state. For butcher stalls, fish markets, slaughter houses, sinks, and wherever there are offensive putrid gasses, sprinkle it about, and in a few days the smell will pass away. If a cat, rat or mouse should die about the house, and send forth an offensive gas, place some chloride of lime in an open vessel near the place where the nuisance is, and it will soon purify the atmosphere. Chloride of lime in a room will cause iron or steel to rust rapidly.

---

GUARANA CHOCOLATE.—This article has been frequently used as a nervous stimulant, and may be drank for a nervous headache, as a restorative in debility, when there is no serious organic disease, in chlorosis, and, indeed, in all affections of a purely nervous character. The taste is agreeable, and as a beverage it is equal to proper chocolate, coffee or tea, if the palate be not too much accustomed to any one kind of drink. The flavor of guarana resembling that of good coffee, persons fond of coffee are likely to be satisfied with this drink. To make the guarana chocolate, take of guarana and white sugar, each, one ounce. Triturate together and then mix thoroughly with good simple chocolate, eighteen ounces. A teaspoonful or more may be dissolved in half a pint of hot water and partaken of at meals as a substitute for coffee or tea.—*Druggists' Circular.*

---

SHAVING SOAP.—To obtain a good soap for shaving is by no means always easy. The great desideratum is to have a soap that makes readily a rich lather which is slow to dry, and that does not require the ceremony of calling for hot water. The most convenient for use are in the shape of paste, so that a little may be taken on the finger and rubbed over the beard; then the brush finishes the process of preparation for the razor. If we take the following ingredients and compound them an excellent soap is produced, that leaves nothing further in this respect to be desired. Take

White soap, 4 oz.

Spermaceti, ½ oz.

Olive oil, ½ oz.

Melt them together, and stir until nearly cold. Scent with such oils as may be most agreeable. Another soap may be made by taking

White wax, }

Spermaceti, } of each, ¼ oz.

Almond oil, }

Melt, and before cooling rub in two cakes of Windsor soap, which have previously been reduced to a paste with a small quantity of rose water. This last, probably, is not unlike a superior shaving soap that has long been in use, and is known as "Rypophagon" soap—a first-rate thing with a very wonderful name.—*Druggists' Circular.*

---

TO DRAW AND PAINT MAGIC LANTERN SLIDES.—They are first prepared by having them out the right size in width and about ten inches in length (they can be bought for a small sum at any glass warehouse); clean them, then lay your picture on a pad of blotting-paper, and place your glass over it; the blotting paper will serve as a bed, and the glass will keep the picture in its place ready for tracing the outline, which is done with a camel's hair paint brush, using ivory black, ground up in the best drying oil, made thin with a little spirits of turpentine. The best outlines are funny men and women, animals, birds, and grotesque figures, sheets of characters, clowns, harlequins, etc. When done in outline with the black, they are fiilled in with the transparent colors, mixed up as the black; only use carmine, gamboge, Prussian blue (the more brilliant the colors, the better effect they produce(, the above being for red, crimson, yellow and blue. To form other transparent colors, mix carmine and Prussian blue for purple, and lavender, gamboge, and Prussian blue for all the shades of green, using for light green more gamboge. Carmine and gamboge make a fine orange color, and for brown shades mix a little ivory black with carmine or lake, with a little gamboge to temper it. Many other tints are made by mixing the primitive colors first named—red, blue and yellow—by using less of one color with another; and if at any time the colors are too thick, thin with turpentine; it works more easily when not too thick, and is more transparent.

When all the colors are finished, mix a nice thin black, and fill in carefully all the ground of the glass round the edges of the figures with the black, leaving no part of the glass slide plain.

These slides should be made very well; and to take better care of them, have them put in small wooden frames, with a tongue at one end to move them in the lantern without the finger touching the glass part. Many beautiful designs can be copied from a kaleidoscope, which, when copied and painted on slides, are very beautiful and show the colors to advantage. Drawing and painting slides is an instructive amusement, and worthy the attention of all persons connected with youth, as it gives them original ideas for combining colors, and thus can be brought into use for many pretty designs in a pleasing manner.—*Scientific American.*

---

Onions sliced, and put in plates, in a sick room, are an excellent disinfectant, and will prevent contagion from eruptive troubles. They should be removed and fresh slices put in their places as soon as discolored. Be sure that these slices are buried, or put where they cannot be eaten, as soon as taken from the room.

It is not safe to use onions that are not taken fresh from the earth, during any epidemic of eruptive diseases, as they are so quickly sensitive to, or impregnated with any contagion or malaria in the atmosphere.—*Christian Union.*

---

### Recipes by Mrs. H. W. Beecher.

CUCUMBER PICKLES.—Gather cucumbers, as above. Put into a stone jar; cover with strong brine—two pounds of salt to five quarts of water. Let them soak twenty-four hours; then drain off the brine and fill up again with boiling water, in which three-fourths of an ounce of alum to every five quarts of boiling water has been dissolved. Put them on the back of range or stove where they will keep warm, but by no means let them boil. Keep them there four or five hours; then pour off all the water and fill the jar with good cider vinegar, as hot as it can be made without boiling. Put cloves, black pepper, mace, etc., unground, into a bag and throw into the vinegar while heating, and leave it in the jar after the vinegar has been poured over the cucumbers. Cover closely and set in a cool dark closet.

TO PICKLE CAULIFLOWERS.—Cut off each cluster from the main head, leaving on as much of the stem as you can. Wash carefully, and for a peck of the clusters sprinkle over a full half pint of salt. Keep them in the salt all night or full twelve hours, when all the salt must be shaken off, taking care not to break the clusters. Throw in a dozen pepper-corns, and cover with scalding hot vinegar. Cover closely, and set aside for use. They will be ready in a few weeks.

PICKLED CABBAGE.—Take solid heads of purple cabbage; remove all the outside leaves; slice from across the head—not up and down—thin, even slices. Put in a large bowl or stone pot one layer of cabbage; then sprinkle salt over it; repeat this with salt; put in another layer about an inch thick; sprinkle on more salt, and so on till the cabbage is all in. Finish with salt on top. Let it stand twenty-four hours. Then to one gallon of best cider vinegar add one ounce each of mace and whole black pepper and a little mustard seed. Drain off all the brine from the cabbage and pack the cabbage into a stone pot or pots, according to the quantity made; have the vinegar and spices scalding hot and pour over the cabbage. Let it stand closely covered till cold; then pour off the vinegar, scald, and again pour over the cabbage. Repeat this three or four times, then cover the jar very tight and put aside in a cool dark place.

GREEN PICKLES.—Put quarter of a pound of salt, two tablespoonfuls of cayenne pepper, one ounce ginger, one ounce white pepper, two ounces of shallots into two quarts of best cider vinegar; boil it a short time, cool, and then pour over any freshly gathered vegetables or fruits which you desire to pickle. Cover closely. Set in a cool, dark place.

CHOCOLATE CARAMELS. — Grate one cup of Baker's chocolate and put it into two cups of boiling milk, add four cups of sugar, stir all thoroughly together, add two cups of best New Orleans molasses, butter size of an egg, one tablespoonful flour, not quite half a teaspoonful soda; put in a clean saucepan or porcelain kettle and boil half an hour. Stir it constantly to prevent scorching. Pour into buttered plates and when cool mark in squares.

Some use water instead of milk, and brown sugar rather than molasses. Just before taking from the fire add any flavor that is preferred. It is usually liked best without any, or a little vanilla.

In making chocolate caramels stir hard till all is dissolved, but after it begins to boil, only shake it to keep from burning. Much stirring will make it grain.

MOLASSES CANDY.—One cup molasses, two cups sugar, one tablespoonful of vinegar, two teaspoonfuls butter, and, if liked, a little vanilla. Boil ten minutes; try by dropping a little in water. If done so as to be brittle, pour into buttered shallow pans, and as soon as cool enough to handle begin to work and pull it.

